# Safety and Efficacy of Cryoballoon Based Pulmonary Vein Isolation in Patients with Atrial Fibrillation and a History of Cancer

**DOI:** 10.3390/jcm10163669

**Published:** 2021-08-19

**Authors:** Charlotte Eitel, Vanessa Sciacca, Nina Bartels, Roza Saraei, Thomas Fink, Ahmad Keelani, André Gaßmann, Karl-Heinz Kuck, Julia Vogler, Christian-Hendrik Heeger, Roland Richard Tilz

**Affiliations:** Department of Cardiology, Angiology, and Intensive Care Medicine, University Hospital Schleswig-Holstein–Campus, 23562 Lübeck, Germany; vsciacca@hdz-nrw.de (V.S.); nikriba@web.de (N.B.); Roza.Saraei@uksh.de (R.S.); tfink@hdz-nrw.de (T.F.); ahmad.keelani@uksh.de (A.K.); andre.gassmann@uksh.de (A.G.); karl-heinz.kuck@uksh.de (K.-H.K.); julia.vogler@uksh.de (J.V.); Christian.heeger@uksh.de (C.-H.H.); roland.tilz@uksh.de (R.R.T.)

**Keywords:** cardiooncology, cryoballoon ablation, atrial fibrillation, cancer, malignoma, pulmonary vein isolation

## Abstract

Introduction: A growing body of evidence suggests a strong association between atrial fibrillation (AF) and cancer. A relevant number of patients with a present or former malignant disease with highly symptomatic drug-refractory AF are in need of interventional therapy. Data on the safety and efficacy of catheter ablation in these patients are sparse. The present study aims to analyze the safety and efficacy of cryoballoon-based pulmonary vein isolation (CB-PVI) for symptomatic AF in patients with past or present cancer disease. Methods and Results: Consecutive patients undergoing CB-PVI for symptomatic AF at University Hospital Lübeck, Germany between July 2015 and January 2019 were included in this study. Propensity-score based matching was performed to identify comparable patients with and without cancer disease and further analyze clinical characteristics, periprocedural complications and arrhythmia-free survival. A total of 70 patients with a history of cancer undergoing CB-PVI were matched to 70 patients without a history of cancer. The frequency of complications was similar between patients with and without a history of cancer (*p* = 0.11), with four phrenic nerve palsies occurring in patients with a history of cancer (5.6% of the cohort) vs. one phrenic nerve palsy in patients without cancer (*p* = 0.36). Arrhythmia free survival after 12 months did not differ significantly in patients with and without a history of cancer (67.1 ± 5.8% vs. 77.8% ± 5.1%, *p* = 0.16). Conclusion: This study indicates that CB-PVI for symptomatic AF is equally safe and effective in patients with and without a history of cancer and cancer treatment.

## 1. Introduction

Effective cancer therapies have improved survival rates, leading to a significant number of cancer survivors who suffer from symptomatic AF. Several studies suggest a strong association between cancer and AF [1,2]. While first reports on the association between AF and cancer showed higher incidences of AF in patients after medical or surgical cancer therapy, recent data indicate a higher prevalence of AF in patients with cancer even before undergoing specific cancer treatment [3]. On the other hand, in patients with new onset AF, the risk of cancer diagnosis was observed to be markedly elevated, especially within the first three months after diagnosis of AF [4,5]. The pathophysiological mechanisms underlying AF in the context of cancer are not fully understood. To date, a multifactorial origin is suspected. Chronic inflammation due to carcinogenesis that leads to oxidative stress, electrical and structural atrial remodeling and thrombogenesis may be relevant factors in initiating AF in cancer patients [6]. Furthermore, paraneoplastic syndromes leading to thyroid disorder or autoimmunity reactions may trigger AF in the context of a malignant disease [7]. Cancer treatment strategies, such as thoracic surgery, radiotherapy, chemotherapy or other pharmaceutical interventions such as steroids, targeted agents or bisphosphonates may also lead to the development of AF [8]. In addition, patients share similar risk factors for either the development of cancer or the development of AF, such as aging, overweight, diabetes, smoking or alcohol consumption. Taking these facts into account, it is obvious that a relevant number of patients experiencing symptomatic AF also have a present or past history of cancer. Today, catheter ablation has become an established therapy for symptomatic patients with AF, with pulmonary vein isolation (PVI) presenting the cornerstone of AF ablation [9]. Different techniques have been developed for safe and effective PVI, with equal rates of success and safety when comparing conventional radiofrequency based and balloon-device based ablation approaches [10]. Until today, it is unclear whether PVI is as effective and safe when performed in patients with present or past cancer diseases. Potential limitations in cancer patients may be procedure- or therapy-related bleeding events or comorbidity, leading to procedure-related complications. Furthermore, cancer or cancer therapy associated atrial fibrosis may necessitate a more extensive ablation approach than PVI only.

The aim of the present study is to evaluate safety and efficacy of cryoballoon-based PVI (CB-PVI) for symptomatic AF in patients with a present or past cancer disease compared to patients without cancer history.

## 2. Materials and Methods

### 2.1. Patient Population

Consecutive patients with symptomatic drug-refractory paroxysmal or persistent AF undergoing CB-PVI at the University Hospital Lübeck, Germany from July 2015–January 2019 were enrolled in this observational study. Baseline, procedural and follow-up data were assessed with special attention on cancer disease and treatment. Cancer disease was defined as a present or past malignant disease requiring surgical, medical and/or radiation therapy. Patients receiving such therapeutical interventions at the timepoint of catheter ablation were defined as patients with active cancer disease. In patients with a history of cancer, telephone interviews were performed to assess details on the specific type of cancer disease, mode of therapy and disease recurrence. The study was in compliance with the principals outlined in the Declaration of Helsinki and approved by the local ethics committee (No. 19-201).

### 2.2. Cryoballoon-Based Pulmonary Vein Isolation

Transesophageal echocardiography was performed in all patients before catheter ablation for exclusion of intracardiac thrombi. All patients then underwent CB-PVI using the 28 mm second or fourth generation cryoballoon (Arctic Front Advance (Pro), Medtronic, Minneapolis, MN, USA). Procedures were conducted in deep sedation with propofol, midazolam and fentanyl. Continuous monitoring of heart rate, blood pressure, oxygen saturation and body temperature was conducted. Heparin was administered after transseptal puncture targeting an activated clotting time of more than 300 s. A diagnostic catheter was advanced via the right femoral vein into the coronary sinus. Esophageal temperature during freezing was monitored with an intraesophageal temperature probe (CIRCA S-CATH, CIRCA Scientific, Englewood, CO, USA). The intraluminal esophageal temperature cut-off was set at 15 °C, leading to the abrupt cessation of further cryoablation. A single transseptal puncture was performed using a modified Brockenbrough technique under fluoroscopic guidance with an 8.5 F transseptal sheath (SL 1, Abbott, North Chicago, IL, USA) and a BRK-1 needle (Abbott). Pulmonary vein ostia were identified by selective pulmonary vein angiography. Over a guidewire, the transseptal sheath was replaced with a 15 F steerable sheath (Flexcath Advance, Medtronic). In all patients, a 20 mm circular mapping catheter (Achieve, Medtronic) was used for guidance of the CB during left atrial movement and for real-time recording of the targeted pulmonary veins. By means of contrast medium injections, complete occlusion by CB was documented. A diagnostic catheter was positioned in the upper caval vein in all patients for phrenic nerve monitoring during freeze application at the septal pulmonary veins. Phrenic nerve pacing was conducted at maximum output and a cycle length of 1000 milliseconds. Capture of the phrenic nerve was detected by tactile feedback of diaphragmatic contraction by the operator, and by monitoring of compound motor action potential. In the case of weakening or loss of diaphragmatic contraction or a compound motor action potential reduction of ≥30%, freeze application was stopped immediately by using a double-stop technique [11].

### 2.3. Ablation Protocol

The standard ablation protocol comprised a fixed freeze-cycle duration of 180 s. Bonus freezes were not applied in a routine manner, but may have been used according to operator’s discretion. If PVI could not be achieved within one freeze-cycle of 180 s, a second freeze-cycle of 180 s was applied. Individual real-time isolation was not taken into account, and the standard freeze-cycle with a 180 s duration was performed. 

### 2.4. Postprocedural Care and Follow-Up

After the ablation procedure, patients were transferred to a waking-up area, where vital sign monitoring was performed for four hours. All patients received transthoracic echocardiography directly after sheath removal, before transmission to the ward and the day after catheter ablation to rule out pericardial effusion. Patients on vitamin K antagonists received uninterrupted anticoagulation with a target INR of two to three. In patients on pre-existing medication with direct oral anticoagulants (DOAC), one dose was paused the morning of the procedure and re-administered 6 h after the procedure. Anticoagulation was continued for at least three months post ablation, and then continued or abandoned based on the CHA_2_DS_2_-VASC Score. Intake of an antiarrhythmic drug therapy was recommended for three months during the blanking period. All patients were administered proton pump inhibitors for 6 weeks after ablation, to reduce the risk of atrioesophageal fistula. The regular scheduled in-hospital stay after ablation was two days. Clinical follow-up was conducted at our outpatient clinic or the referring physician after 3, 6 and 12 months, including assessment of the clinical history, 12-lead ECG and 24 h Holter ECG. Device interrogation was conducted in patients with implantable cardiac devices.

### 2.5. Definition of Complications and Cancer Activity

All periprocedural complications were documented and analyzed. Complications were defined as periprocedural when occurring intraprocedural, postprocedural during the hospital stay or until 30 days after the procedure. Complications were classified as major complications if permanent injury, interventional treatment, prolonged hospital stay, repeat hospitalization for more than 48 h or death occurred, as described in the consensus statement for catheter ablation of AF [12]. Bleeding complications were classified as described in the criteria of the International Society on Thrombosis and Hemostasis as major bleeding or clinically relevant non-major bleeding [13].

Cancer disease was defined as active in patients receiving any kind of specific anti-cancer treatment when undergoing ablation, such as radiation, chemotherapy or oral medication such as selective estrogen receptor modulators, aromatase inhibitors, antiandrogen medication or antibody therapy. Patients without active cancer disease at the timepoint of ablation but a history of cancer were defined as cancer survivors.

### 2.6. Statistical Analysis

Categorical data were referred to as frequencies and continuous data as mean ± standard deviation (SD). Categorical data were further analyzed and compared by implementation of the χ2-test or Fisher’s exact test. Continuous data are expressed as median with quartiles and analyzed by the use of the nonparametric Wilcoxon rank sum. *p*-values were based on two-sided testing and values <0.05 were considered to be statistically significant. Survival curves of arrhythmia-free survival after ablation were estimated with the Kaplan–Meier using SPSS (IBM, Armonk, NY, USA; version 26).

To define statistical twins without cancer disease for each patient with cancer disease, a propensity score matching (PSM) with fuzzy logic was performed on SPSS with three different matching tolerances (0.2, 0.5 and 1.0). Age, sex, CHA2DS2-VASC score, type of AF (persistent or paroxysmal AF) and duration of follow-up were chosen as predictors. A tolerance of 0.5 was accepted for adequate comparability of both sample cohorts, resulting in a 1:1 matching.

## 3. Results

### 3.1. Baseline Characteristics

After propensity-score based matching 70 patients with and without present or past cancer disease undergoing CB-PVI were analyzed (central illustration). Of these 55.7% were male with a mean age of 70.5 ± 8.5 years. The majority of patients (76.9%) had persistent AF and median CHA_2_DS_2_-VASC score was 3 (IQR 2;4). All patients were on a regular medication with oral anticoagulants with 111 patients (79.3%) receiving DOAC and 29 patients (20.1%) receiving vitamin K antagonists. There were no significant differences with respect to baseline characteristics in patients with and without cancer. Details on baseline characteristics are shown in Table 1.

### 3.2. Specific Disease Related Characteristics in Patients with a History of Cancer

The majority of patients with cancer were cancer survivors who did not suffer from active cancer when undergoing ablation (62 of 70 patients, 88.6%). In eight patients (11.4%), an active cancer disease was present when ablation was performed. Regarding cancer entities, the most frequent cancer observed in this cohort was genitourinary cancer (30%), followed by breast cancer (28.6%), haemato-oncologic cancer (12.9%), gastrointestinal cancer (11.4%), head or neck cancer (5.7%) and lung cancer (2.9%). A history of more than one cancer entity was observed in 8.6% of the patients. Specifically, one patient had a history of breast and lung cancer; one patient had a history of breast and endometrium cancer; one male patient had a history of breast cancer, testicular cancer and laryngeal cancer; two patients suffered from genitourinary cancer and gastrointestinal cancer; and one patient from prostate and bladder cancer.

In 50 patients (71.4%), radiation was performed for cancer treatment with 29 of these patients (56%) undergoing thoracic radiation. In 22 patients (31.4%), chemotherapy was conducted. In 18 patients (25.7%), a combined radio-chemotherapy was conducted for initial cancer treatment. Antihormone therapy was conducted in 19 patients (27.1%) and antibody therapy in 4 patients (5.7%). Within the subgroup of eight patients with active cancer at the timepoint of ablation, the present treatment strategy was antihormone therapy in six patients, radiation in one patient and a tyrosin kinase inhibitor in one patient.

### 3.3. Procedural Data

Mean procedure time was 128.7 ± 36.1 min, and mean fluoroscopy time was 20.3 ± 9.9 min, with statistically longer procedure duration and fluoroscopy time in patients without cancer. Furthermore, real-time isolation of right pulmonary veins and left inferior pulmonary vein were significantly more often achieved in patients without cancer disease. Details are shown in Table 2.

### 3.4. Periprocedural Complications

Regarding major procedure related complications, there was one phrenic nerve palsy (PNP) in the group of patients without a history of cancer and four PNPs in patients with a history of cancer (1.4% vs. 5.6%, *p* = 0.36). With respect to minor periprocedural complications, femoral pseudoaneurysm occurred in two patients (2.9%) with a history of cancer, in one patient (1.4%) without a history of cancer (*p* = 1.0) and could be treated conservatively in all. In both cohorts, no periprocedural cardiac tamponade, relevant bleeding, atrioesophageal fistula or death occurred.

### 3.5. Arrhythmia Follow-Up and Repeat Ablation Procedures

Mean follow-up duration in the cancer cohort was 606.7 ± 350.8 days and 670.4 ± 396.6 days in the non-cancer cohort. Arrhythmia recurrence after single CB-PVI outside of the blanking period occurred in 32 patients (45.7%) with a history of cancer (AF in 27 patients (84.4%) and atrial tachycardia (AT) in five patients (15.6%)) versus 26 patients (37.1%) without a history of cancer (AF in 21 patients (80.8%), AT in four patients (15.4%) and common type atrial flutter in one patient (3.8%)). 

Repeat ablation procedures were performed in 8 patients (11.4%) in the cancer group and in 16 patients (22.9%) in the non-cancer group (*p* = 0.11) (Table 3). Indication for repeat ablation was AF in two (25%) versus nine (56.3%) patients in the cancer and non-cancer group (*p* = 0.23), and AT in six (75%) versus seven patients (43.8%), respectively (*p* = 0.23). 

At the timepoint of the last follow-up, including repeat ablation procedures, 56 patients (80%) with a history of cancer were in sinus rhythm, while 14 patients (20%) presented with AF. Within the group of patients without a history of cancer, 58 patients (82.9%) were in sinus rhythm, while 8 patients (11.4%) presented with AF and 4 patients (5.7%) with AT.

### 3.6. Estimation of Arrhythmia-Free Survival

**Figure 1:**
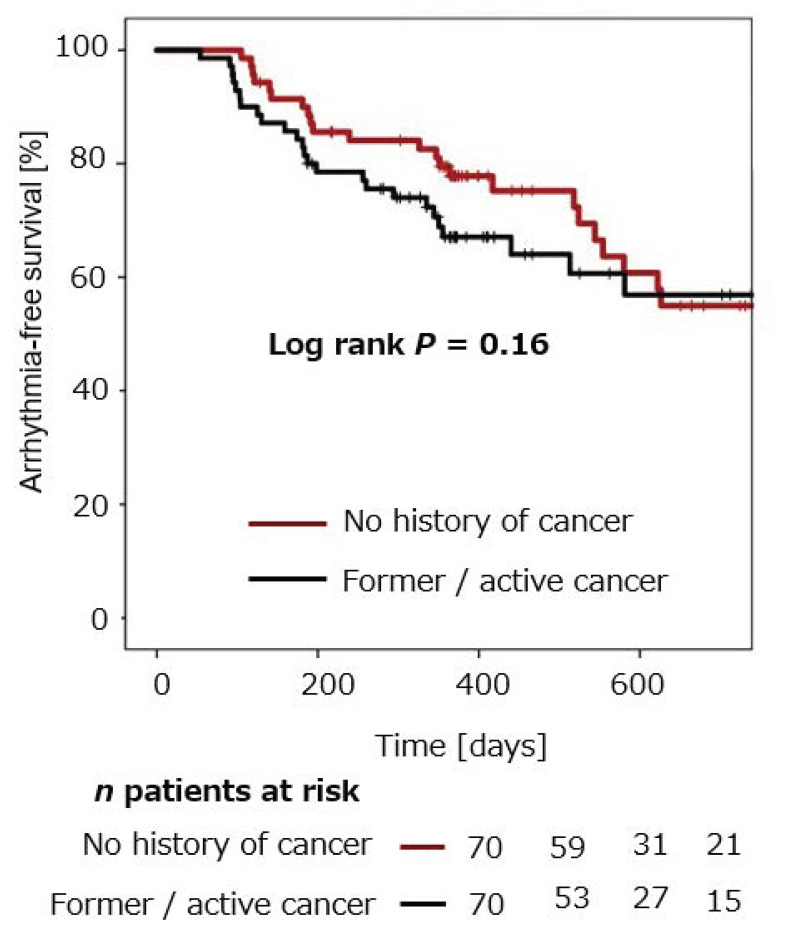
Cumulative freedom from arrhythmia recurrence in patients with and without a history of cancer after CB-PVI.

Estimation of arrhythmia-free survival after CB-PVI within both groups was performed using the Kaplan–Meier method. In patients without a history of cancer, mean arrhythmia-free survival after single CB-PVI was 77.8% ± 5.1% after 12 months and 55.0% ± 7.7% after 24 months. Mean arrhythmia-free survival in patients with a history of cancer was 67.1 ± 5.8% after 12 months and 56.9% ± 7.4% after 24 months (Figure 1). No statistically significant difference in arrhythmia recurrence was observed in the two groups (*p* = 0.16). 

Patients with a history of cancer were estimated to be free from arrhythmia recurrence in 67.1 ± 5.8% after 12 months. Patients without a history of cancer were estimated to be free from arrhythmia recurrence after 12 months in 77.8% ± 5.1%. There was no statistically significant difference in estimated freedom from arrhythmia recurrence in patients with and without a history of cancer following CB-PVI (log rank *p* = 0.16).

**Figure 2:**
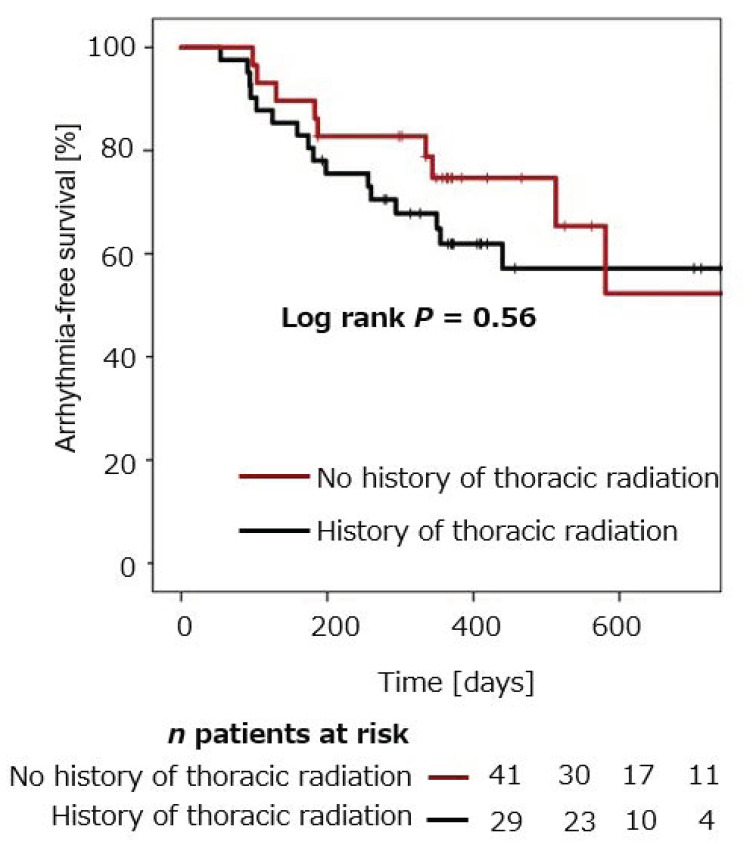
Cumulative freedom from arrhythmia recurrence after CB-PVI in cancer patients with thoracic radiation compared to cancer patients without thoracic radiation.

Arrhythmia-free survival was compared in patients with cancer and thoracic radiation (*n* = 29), and compared to patients with cancer and without thoracic radiation (*n* = 41). Mean arrhythmia-free survival in cancer patients with versus without thoracic radiation was 74.7 ± 8.3% versus 61.9 ± 7.8% after 12 months, and 52.3 ± 14.8% versus 57.1 ± 8.6% after 24 months (*p* = 0.56), respectively (Figure 2).

Mean arrhythmia-free survival in cancer patients with former thoracic radiation was 74.7 ± 8.3% after 12 months. Mean arrhythmia-free survival in cancer patients without former thoracic radiation was 61.9.1 ± 7.8% after 12 months. No significant difference was observed between the two groups (log rank *p* = 0.56). 

Arrhythmia-free survival did not differ significantly in cancer patients who received chemotherapy (31.4%) compared to cancer patients who did not receive chemotherapy (*p* = 0.61). In cancer patients who received antibody therapy (27.1%), no significant difference in arrhythmia recurrence rates compared to cancer patients without antibody therapy was observed either (*p* = 0.39).

Arrhythmia recurrence rate in patients with cancer was further analyzed regarding gender. No gender related differences in arrhythmia recurrence were observed (*p* = 0.93).

## 4. Discussion

To the best of our knowledge, the present study assesses the largest cohort of patients with a history of cancer undergoing catheter ablation for symptomatic AF. More specifically, this is the first study analyzing the safety and efficacy of CB-PVI in patients with a history of cancer compared to a matched cohort of patients without. We found that (1) patients with a history of cancer had a similar complication rate as patients without cancer; (2) CB-PVI resulted in arrhythmia-free survival in a high number of patients with paroxysmal and persistent AF, without significant differences between patients with and without cancer history and (3) arrhythmia-free survival did not differ in patients with and without previous radiotherapy for cancer disease.

### 4.1. Atrial Fibrillation Ablation in Patients with Cancer Disease

Frequency of cancer in patients with AF has been observed in up to 17% of patients [14]. Previous studies showed that even many years after surviving cancer the cardiovascular mortality is significantly elevated, in comparison to people without a history of cancer [15]. Due to improvements in cancer therapies with longer survival after cancer diagnosis, the number of patients with AF and cancer is expected to further increase with a relevant proportion of patients in need of interventional therapy for symptomatic AF. Our study shows similar arrhythmia-free survival rates without significant differences in periprocedural complications in patients with a history of cancer compared to patients without. In contrast, a recently published study by Giustozzi and coworkers found higher periprocedural bleeding rates in cancer survivors undergoing catheter ablation for AF [16]. This discrepancy may be explained by several facts: First, in the above-mentioned study, patients underwent only radiofrequency-based ablation, potentially resulting in a longer procedure duration with the administration of higher doses of heparin intraprocedurally, possibly leading to increased bleeding events. Second, patients in the study by Giustozzi were on interrupted anticoagulation bridged with low molecular weight heparin when ablation was conducted, whilst all patients in the present cohort received oral anticoagulation without interruption. This finding is in line with previous studies, indicating higher periprocedural bleeding rates in patients receiving bridging with low molecular weight heparin post ablation [17]. Our results suggest that periprocedural anticoagulation with the administration of therapeutic dosages of heparin with a target ACT of 250–300 are safe in cancer patients. However, the number of patients with active cancer disease was low, limiting transferability of our results to this specific patient cohort. 

Statistical differences between the two groups in longer procedure duration and fluoroscopy time in patients without cancer may be caused by several facts: (1) more efforts to shorten procedure time in patients with assumed higher procedural risk, due to comorbidities and cancer; (2) stronger attempts to record real-time signals of PVI in patients with assumed lower procedural risk and without cancer; (3) by chance, a higher number of patients had left common ostium without cancer disease. 

With respect to arrhythmia recurrences, there were no significant differences in the occurrence of AF or AT as a mode of arrhythmia recurrence in patients with and without cancer history. However, patients with a history of cancer underwent repeat ablation less frequently (11% of cancer patients versus 22.9% of patients without cancer history), and indication for repeat ablation with AT in 75% of cancer patients versus 43.6% in patients without cancer. This can probably be attributed to the fact that the indication for catheter ablation is stricter in patients with comorbidities, such as cancer, and that AT can hardly be treated by conservative therapies and medical treatment, finally strengthening the indication for repeat ablation. 

Overall, these results indicate that CB-PVI is a feasible and safe treatment option for symptomatic AF in patients with cancer disease. This finding is of importance, as CB-PVI has shown an excellent safety and efficacy profile, and could be a preferred option in patients with a marked comorbidity, such as prior cancer disease [18].

### 4.2. Cryoballoon Ablation in Patients with Previous Thoracic Radiation

Radiotherapy of the chest can induce cardiac inflammation and potentially fibrosis, potentially leading to higher incidences of AF and AT [19]. Therefore, patients with former chest radiation may exhibit more left atrial fibrosis, along with a higher rate of organized AT. One might suggest that these patients would necessitate a more extensive ablation approach than a pure CB-PVI. Of note, the present study showed comparable arrhythmia-free survival after CB-PVI in patients who underwent thoracic radiation, indicating feasibility of CB-PVI in these patients. Our finding might be in line with a more recent study, showing that thoracic irradiation and chemotherapy in a collective of patients with former breast cancer did not lead to an increase in left atrial scarring or a different distribution of left atrial scar distribution, based on 3D left atrial voltage mapping [20].

### 4.3. Limitations

The number of patients with cancer was relatively low, with the majority of patients being cancer survivors and only a small number of patients having active cancer disease at the timepoint of ablation. Furthermore, the collective was heterogeneous regarding type of cancer and cancer treatment. In the present study, the impact of thoracic radiation on the clinical outcome of AF ablation was analyzed in detail. The impact of other cancer therapies, such as chemotherapy or antibody therapy, needs detailed investigation in larger studies. Nevertheless, to our knowledge, the present study analyzed the largest cohort of patients with cancer disease undergoing CB-PVI. The results presented in this study need to be evaluated further in larger cohorts, especially including more patients with an active cancer disease. 

## 5. Conclusions

In the present study, high arrhythmia-free survival with low frequencies of periprocedural complications were observed in patients with a history of cancer undergoing CB-PVI. Procedural safety and arrhythmia-free survival was comparable to patients without cancer disease, leading to the conclusion that CB-PVI is a feasible therapy in this special patient cohort.

## Figures and Tables

**Table 1:** Baseline characteristics of patients with and without cancer history.

	No Cancer *N* = 70	Cancer *N* = 70	*p*-Value
Age (years); mean ± SD	69.7 ± 8.7	71.3 ± 8.3	0.27
Sex, male; *n* (%)	39 (55.7)	39 (55.7)	1.00
BMI, kg/m^2^; mean ± SD	28.0 ± 6.5	27.6 ± 5.9	0.70
Paroxysmal AF, *n* (%)	23 (32.9)	22 (31.4)	0.86
Persistent AF, *n* (%)	47 (67.1)	48 (68.6)	0.86
CHA2DS2-VASC score; median (IQR)	3 (2;4)	3 (2;4)	0.15
Oral anticoagulation with DOAC, *n* (%)	55 (78.6)	56 (80)	1.0
Oral anticoagulation with vitamin K antagonist, *n* (%)	15 (21.4%)	14 (20)	1.0
Medical History			
Arterial Hypertension; *n* (%)	55 (78.6)	49 (70)	0.33
Coronary Artery Disease; *n* (%)	10 (14.3)	12 (17.1)	0.81
Chronic Renal Insufficiency; *n* (%)	17 (24.3)	22 (31.4)	0.45
Diabetes mellitus	7 (10)	6 (8.6)	1.00
Implanted cardiac device (%)	11 (15.7)	12 (17.1)	1.00
LVEF (%); mean ± SD	52.2 ± 8.7	50.4 ± 11.6	0.30

AF = atrial fibrillation; BMI = body mass index; IQR = interquartile range; LVEF = left ventricular ejection fraction; SD = standard deviation.

**Table 2:** Procedural characteristics and complications of patients with and without cancer history.

	No Cancer *N* = 70	Cancer *N* = 70	*p*-Value
Procedure duration (min); mean ± SD	137.9 ± 27.3	116.4 ± 42.4	<0.001
Fluoroscopy time (min); mean ± SD	24.8 ± 8.5	18.2 ± 9.8	<0.001
Number of freezes RSPV; mean ± SD	1.3 ± 0.6	1.4 ± 0.8	0.40
Number of freezes RIPV; mean ± SD	1.4 ± 0.6	1.4 ± 0.6	1.00
Number of freezes LSPV; mean ± SD	1.5 ± 0.7	1.4 ± 0.5	0.33
Number of freezes LIPV; mean ± SD	1.3 ± 0.5	1.4 ± 0.7	0.33
Common left ostium; *n* (%)	11 (15.7)	4 (5.7)	0.10
Real time isolation RSPV; *n* (%)	49 (70)	29 (41.4)	0.001
Real time isolation RIPV; *n* (%)	45 (64.3)	15 (21.4)	<0.001
Real time isolation LSPV; *n* (%)	31 (44.3)	29 (41.4)	0.86
Real time isolation LIPV; *n* (%)	43 (61.4)	25 (35.7)	0.004
Freezing time RSPV (s); mean ± SD	249.9 ± 136.8	223.1 ± 94.5	0.18
Freezing time RIPV (s); mean ± SD	291.1 ± 151.9	278.9 ± 136.3	0.62
Freezing time LSPV (s); mean ± SD	304.1 ± 153.5	250.9 ± 104.6	0.02
Freezing time LIPV (s); mean ± SD	266.8 ± 122.1	260.9 ± 136.8	0.79
Complications			
Phrenic nerve palsy, *n* (%)	1 (1.4)	4 (5.7)	0.36
Pseudoaneurysm, *n* (%)	1 (1.4)	2 (2.8)	1.0

LSPV = left superior pulmonary vein; LIPV = left inferior pulmonary vein; RSPV = right superior pulmonary vein; RIPV = right inferior pulmonary vein; SD = standard deviation.

**Table 3:** Arrhythmia follow-up of patients with and without history of cancer.

	No Cancer *N* = 70	Cancer *N* = 70	*p*-Value
Arrhythmia recurrence, *n* (%)	26 (37.1)	32 (45.7)	0.23
Mode of arrhythmia recurrence			
AF, *n* (%)	21 (80.8)	27 (84.4)	0.92
AT, *n* (%)	4 (15.4)	5 (15.6)	0.92
Repeat ablation, *n* (%)	16 (22.9)	8 (11.4)	0.11
AF at repeat procedure, *n* (%)	9 (56.3)	2 (25)	0.23
AT at repeat procedure, *n* (%)	7 (43.8)	6 (75)	0.23

## Data Availability

The data that support the findings of this study are available from the corresponding author (C.E.), upon reasonable request. This author takes responsibility for all aspects of the reliability and freedom from bias of the data presented and their discussed interpretation.

## Abbreviations

AF	Atrial Fibrillation
AT	atrial tachycardia
BMI	body mass index
CB-PVI	cryoballoon-based pulmonary vein isolation
LSPV	left superior pulmonary vein
LIPV	left inferior pulmonary vein
PNP	phrenic nerve palsy
PVI	pulmonary vein isolation
RSPV	right superior pulmonary vein
RIPV	right inferior pulmonary vein
